# Tuning Water Networks via Ionic Liquid/Water Mixtures

**DOI:** 10.3390/ijms21020403

**Published:** 2020-01-08

**Authors:** Archana Verma, John P. Stoppelman, Jesse G. McDaniel

**Affiliations:** Georgia Institute of Technology, School of Chemistry and Biochemistry, Atlanta 30332-0400, Georgia; archana.x.verma@gmail.com (A.V.); jstoppelman3@gatech.edu (J.P.S.)

**Keywords:** ionic liquids, molecular dynamics, percolation network, ab initio force fields, water mixtures, nanoconfinement

## Abstract

Water in nanoconfinement is ubiquitous in biological systems and membrane materials, with altered properties that significantly influence the surrounding system. In this work, we show how ionic liquid (IL)/water mixtures can be tuned to create water environments that resemble nanoconfined systems. We utilize molecular dynamics simulations employing *ab initio* force fields to extensively characterize the water structure within five different IL/water mixtures: [BMIM${}^{+}$][BF${}_{4}^{-}$], [BMIM${}^{+}$][PF${}_{6}^{-}$], [BMIM${}^{+}$][OTf${}^{-}$], [BMIM${}^{+}$][NO${}_{3}^{-}$] and [BMIM${}^{+}$][TFSI${}^{-}$] ILs at varying water fraction. We characterize water clustering, hydrogen bonding, water orientation, pairwise correlation functions and percolation networks as a function of water content and IL type. The nature of the water nanostructure is significantly tuned by changing the hydrophobicity of the IL and sensitively depends on water content. In hydrophobic ILs such as [BMIM${}^{+}$][PF${}_{6}^{-}$], significant water clustering leads to dynamic formation of water pockets that can appear similar to those formed within reverse micelles. Furthermore, rotational relaxation times of water molecules in supersaturated hydrophobic IL/water mixtures indicate the close-connection with nanoconfined systems, as they are quantitatively similar to water relaxation in previously characterized lyotropic liquid crystals. We expect that this physical insight will lead to better design principles for incorporation of ILs into membrane materials to tune water nanostructure.

## 1. Introduction

Water is a key component of numerous chemical, biological and geological systems and materials. The properties of water-containing systems are strongly influenced by water’s unique hydrogen bonding structure and large cohesive energy relative to its molecular weight, with these attributes giving rise to the universally important hydrophobic effect [1]. Likewise, the surrounding chemical environment may perturb the structure and dynamic properties of water itself, which is particularly significant when water exists in nanoconfinement. Examples of water in nanoconfinement are ubiquitous, including water in biological and/or artificial membranes, lyotropic liquid crystals, cavities in proteins and enzymes, carbon nanotubes, zeolites and metal organic frameworks [2,3,4,5,6,7]. In nanoconfinement, a substantial fraction of water is proximal to an interface, and the geometry and chemical functionality of the interface modulates the hydrogen bond network, which under certain conditions prevents water from freezing [8]. Beyond academic interest, understanding the behavior of water in strongly perturbed environments has technological importance in membrane science, catalysis, electrochemistry, separations and medicine.

An illustrative example of the technological importance of nanoconfined water is within the context of proton exchange membrane (PEM) materials, which are an essential component of fuel cells [9]. Perfluorinated sulfonic acid (PFSA) membranes including Nafion [3] are widely utilized PEM materials, which exhibit hydrophilic water pockets of several nanometers in diameter connected by nanometer-sized aqueous channels [3,10]. The structure, dynamics and percolation of these nanoconfined water pockets and networks largely dictate the PEM performance (e.g., proton conductivity), as proton transport and diffusion occurs primarily through the Grotthuss hopping mechanism [11,12,13]. In Grotthuss hopping, protons migrate along water networks via rearrangement of covalent and hydrogen bonds, and the efficiency of this process intrinsically depends on the structural connectivity of water; for example, 1D water “wires” such as found in carbon nanotubes may exhibit order(s) of magnitude enhanced proton diffusion rates due to their unique water topology [14]. Thus the ability to tune the nanostructure of water in membrane materials would provide an important design parameter for enhancing performance and ion transport.

Ionic liquids (ILs) [15,16,17,18,19,20,21,22] as solvents provide a unique environment capable of tuning the connectivity and structure of water over nanometer lengthscales. This is because the organic molecular ions that comprise ILs exhibit both hydrophilic and hydrophobic interaction motifs; while water favorably interacts with the dipolar functional groups and ionic charges, aliphatic chains on typical IL cations and/or -CF${}_{3}$ groups on anions give rise to hydrophobic effects and promote water clustering and aggregation. IL/water mixtures thus span the entire spectrum from fully mixed and miscible systems, to phase separated IL and water domains, with varying motifs of water structure and clustering in between [16,23,24,25,26,27]. Unlike typical surfactant-based systems in which water phase behavior is largely modulated by the volume fraction of surfactant aliphatic groups, ILs modulate water structure in absence of significant changes in ionic/hydrophobic volume ratio. For example, in ILs composed of 1-butyl-3-methylimidazolium (BMIM${}^{+}$) cations, switching from similar-sized tetrafluoroborate (BF${}_{4}^{-}$) to hexafluorophosphate (PF${}_{6}^{-}$) anions qualitatively changes IL/water mixtures from fully-miscible and hydrophilic, to immiscible (hydrophobic) and phase-separated IL and water domains! This dramatic variation in IL/water miscibility from different anions is due to electrostatic screening energetics arising from the intrinsic electrostatic properties of the bulk ILs themselves [28]. In addition to the significant benefit of enhanced thermal stability, the ability to tune the water/ion nanostructure has motivated the incorporation of ILs into Nafion and/or other PFSA-like PEMs to potentially enhance performance [29,30,31,32,33].

In addition to membranes, other potential applications of IL/water mixtures include use as solvents for chemical extraction, separation and catalysis [16,24,26,34,35], solvents for biomolecules and protein stabilizers [36,37,38,39,40], tunable solvents for biocatalysis [41,42,43,44] and solvents for tailoring coordination chemistry and exchange rates of lanthanides [45,46]. In all of these applications, it is essential to develop a fundamental understanding of how different ILs alter water nanostructure at various ion/water concentrations. Based on intrinsic hydrophobicity differences among ILs, it is expected that different ILs will yield a wide variety of nanostructured water motifs including wires, clusters, water pockets or percolation networks. The ability to modify water nanostructure with different ILs presents an exciting possibility for tuning mixtures but requires substantial understanding of the physical properties of ILs for predictive design. For example, even miscibility trends are difficult to rationalize for certain ILs: Despite the similar nature of the BF${}_{4}^{-}$ and PF${}_{6}^{-}$ anions, [BMIM${}^{+}$][BF${}_{4}^{-}$] is a hydrophilic IL and fully miscible with water, while [BMIM${}^{+}$][PF${}_{6}^{-}$] is hydrophobic and phase separates at significant water content. While rationalization of miscibility trends have primarily focused on the nature of the anion [23,24,47], recent work suggests that miscibility may depend on the net electrostatic interactions within the bulk IL resulting from a synergy of cation and anion contributions [28]. Understanding the physical principles dictating these trends is essential to enable tuning of water networks within IL/water mixtures and requires comprehensive investigation of a variety of systems and not just studies on a case-by-case basis.

Experimental spectroscopy [25,48,49,50,51,52,53,54,55,56,57], small angle X-ray or neutron diffraction (SAXS/SANS) [58,59,60,61,62,63,64,65], and nuclear magnetic resonance (NMR) studies [66,67,68,69,70,71] have revealed a wealth of information on the structural and dynamic properties of IL/water mixtures. We restrict such synopsis to ILs composed of shorter chain cations similar to BMIM${}^{+}$; it is well-documented that ILs composed of longer chain cations form micelles, reverse micelles or self-assemble when mixed with water [53,58,60,72,73,74] but this surfactant-like regime is not our focus. Vibrational red shifts of water indicate how hydrogen bonding/structure is altered by local environment and have been characterized in a variety of ILs at dilute water concentrations [25,52] and as a function of water content [50,56]. Under dilute conditions, spectroscopic measurements indicate that water molecules interact primarily with the anions within both hydrophobic and hydrophilic ILs [49,51]. At higher water concentration within hydrophilic IL/water mixtures, a number of different peaks were observed for the water stretch (deuterated) [50], with relative populations being significantly concentration dependent and suggesting the formation of water domains or clusters [56]. NMR [69] and SAXS/SANS experiments [61,63,65] have independently led to the same conclusion that water within hydrophilic ILs may resemble water states in nanoconfined systems. SAXS/SANS experiments indicate the formation of water pockets within hydrophilic BMIM${}^{+}$/nitrate ([BMIM${}^{+}$][NO${}_{3}^{-}$]) ILs that are similar to those formed within nanoconfined systems [61], with the size of water pockets modulated by water concentration [65] and exhibiting anomalous temperature behavior [63]. In hydrophilic [BMIM${}^{+}$][BF${}_{4}^{-}$]/water mixtures, NMR measurements show substantial changes in ion diffusion trends between 0.2 to 0.4 water mole fraction, indicating changes in microscopic structure [67]. Structural heterogeneity and microscopic domain formation occurs in hydrophobic IL/water mixtures, as indicated for BMIM${}^{+}$/bis(trifluoromethylsulfonyl)imide ([BMIM${}^{+}$][TFSI${}^{-}$]) based on NMR measurements [66]. There exists disagreement, however, on the lengthscale of such water pockets or confined microdomains within ILs; some studies report lengthscales on the order of several nanometers [61], while other studies suggest that domains are formed from fewer than ∼5 water molecules [71]. This question has been stated in recent work simply as whether “water molecules in IL matrices (are) dispersed or does a water pool form?” [71]. We note that experimental characterization of changes in transport properties (e.g., conductivity, viscosity) of ILs with water content can provide clues about internal water structure [23,27,47,75,76,77,78] but such analysis is indirect and requires care [79].

Complementing experimental studies, molecular dynamics (MD) simulations have provided much insight into the structure of IL/water mixtures. Water clustering in IL/water mixtures has been studied for [BMIM${}^{+}$][BF${}_{4}^{-}$] and 1-ethyl-3-methylimidazolium/BF${}_{4}^{-}$ ([EMIM${}^{+}$][BF${}_{4}^{-}$]) [80,81,82,83,84,85,86,87], 1,3-dimethylimidazolium (DMIM${}^{+}$)/halides and BMIM${}^{+}$/halides [88,89,90,91], EMIM${}^{+}$/acetate ([EMIM${}^{+}$][CH${}_{3}$COO${}^{-}$]) [91,92,93], [EMIM${}^{+}$][TFSI${}^{-}$] [80], [BMIM${}^{+}$][PF${}_{6}^{-}$] [90] and ILs with longer aliphatic chains (which are more “surfactant-like”) [72,73,82,94,95]. However, only some of these studies have systematically analyzed concentration-dependent trends. For [BMIM${}^{+}$][BF${}_{4}^{-}$]/water mixtures, it was found that significant water clustering began to occur at concentrations above 0.4 mole fraction water [82,83]. Niazi et al. predicted very little water clustering in chloride and acetate-based IL/water mixtures even at moderate water content, followed by a stepwise “jump” in cluster growth at ∼70% mole fraction [91]. Another study rationalized the significantly reduced water clustering in [EMIM${}^{+}$][CH${}_{3}$COO${}^{-}$] compared to [EMIM${}^{+}$][BF${}_{4}$] IL/water mixtures as due to formation of anion-water-anion wire structural motifs in the acetate IL [93]. A number of simulation studies have examined IL/water liquid/liquid [96,97,98], liquid/vapor [99] or other interfaces [100], with other studies focused on predicting thermodynamic properties of IL/water mixtures, with structural characterization not being the primary focus [28,101,102,103,104,105,106,107]. Structural predictions from simulations may be validated through spectroscopic connection with experiment but such “first-principles” condensed phase spectroscopic predictions for IL/water mixtures are rare [108].

The motivation for the present work is to provide a systematic analysis of water structure in IL/water mixtures for a number of different ILs at varying water concentration by employing molecular simulations that utilize highly accurate force fields. Such trends may be difficult to infer from the collection of previously cited work, due to the high sensitivity of predicted water clustering/phase behavior to force field details [96,97,98,106]. McDaniel and coworkers have recently developed the *ab initio*, polarizable SAPT-FF force field for a variety of ILs [109,110], with property prediction benchmarks for neat ILs [111] as well as a variety of IL solvent mixtures [112]. For IL/water mixtures, the force field employs an *ab initio* description of cross-interactions [113] and water-water interactions are described by the well-benchmarked SWM4-NDP water model [114]. Explicit polarization is expected to be important for accurate predictions for two primary reasons. First, polarization has a universal and systematic influence on the structure of neat ionic liquids due to fundamental screening conditions [111,115] and this effect is expected to modulate the structure in low water content IL systems. Additionally, it is expected that polarization is important for accurately describing IL/water interfaces and thus phase behavior in hydrophobic IL mixtures [96,97,98].

In this work, we use molecular dynamics simulations to investigate water structure in five different IL/water mixtures with hydrophilic [BMIM${}^{+}$][BF${}_{4}^{-}$], [BMIM${}^{+}$][NO${}_{3}^{-}$] and BMIM${}^{+}$/triflate ([BMIM${}^{+}$][OTf${}^{-}$]) ILs and hydrophobic [BMIM${}^{+}$][PF${}_{6}^{-}$] and [BMIM${}^{+}$][TFSI${}^{-}$]. We demonstrate that water nanostructure can be sensitively tuned by choice of IL. Hydrophilic IL/water mixtures generally exhibit diffuse water molecules and small clusters at low water content, while hydrophobic ILs exhibit water pockets near their saturation limit. Percolating water networks form in the hydrophilic IL mixtures at ∼15–25% water by volume and [BMIM${}^{+}$][OTf${}^{-}$] is the best hydrophilic IL for facilitating water percolation due to the amphiphilic nature of the triflate anion. In addition to this structural characterization, we show that water rotational relaxation times are quite similar to those in a variety of lyotropic liquid crystals, supporting the notion that water in IL/water mixtures is reminiscent of water in nanoconfinement. We anticipate that this physical insight will be important for the application of IL/water mixtures within membrane materials.

## 2. Methods

Molecular dynamics (MD) simulations were conducted for [BMIM${}^{+}$][BF${}_{4}^{-}$], [BMIM${}^{+}$][PF${}_{6}^{-}$], [BMIM${}^{+}$][NO${}_{3}^{-}$], [BMIM${}^{+}$][OTf${}^{-}$] and [BMIM${}^{+}$][TFSI${}^{-}$]/water mixtures at systematically varying water volume fraction of $\phi_{H_{2}O}$ = 0.02, 0.04, 0.08, 0.13, 0.19 and 0.25. To enable simulation box sizes of at least 4–5 nanometers, all simulations employed 220 ion pairs and the number of water molecules was chosen to give the desired water volume fraction, $\phi_{H_{2}O}$; the specific number of water molecules corresponding to the IL/water volume fraction mixtures is given in Table 1. All IL/water simulations were conducted at 300 K, 1 bar, except for [BMIM${}^{+}$][NO${}_{3}^{-}$]/water mixtures which were run at 320 K so as to be sufficiently above the melting point of the IL. The IL/water mixtures were modeled utilizing the SAPT-FF force field for IL/IL and IL/water interactions [111,113] and SWM4-NDP water model [114] for water/water interactions. This explicitly polarizable, force field combination has been previously demonstrated to predict accurate conductivities of IL/water mixtures [112] and excess chemical potentials of water in ILs [28].

MD simulations were conducted using the OpenMM software package [116], on Nvidia GTX-1080-Ti GPU cards. Initial systems were prepared using the Packmol software [117] and equilibrated in the NPT ensemble for 10 ns, and production runs were propagated for 20 ns in the NVT ensemble. The simulations were run with a 1 fs timestep and simulation frames were saved every 1 ps for analysis. A dual Langevin thermostat was utilized for temperature coupling of both real and Drude oscillator degrees of freedom [118], with friction coefficients of 1 ps${}^{-1}$ for both thermostats and fictitious mass of 0.4 amu on the Drude particles. Electrostatic interactions were computed with the particle mesh Ewald (PME) approach [119] and van der Waals interactions were truncated at 1.4 nm accompanied with standard long-range correction. The MDTraj software was utilized for simulation analysis [120], NetworkX software was used for clustering analysis [121] and a recursive algorithm was employed for percolation analysis [122]; specific details of the analysis methods are discussed in the Supporting Information.

We comment on our choice of water content range for studying hydrophobic [BMIM${}^{+}$][PF${}_{6}^{-}$] and [BMIM${}^{+}$][TFSI${}^{-}$] IL/water mixtures. Experimentally, hydrophobic ILs generally absorb several percent water fraction by volume before phase separating into water-rich and IL-rich phases; for example [BMIM${}^{+}$][PF${}_{6}^{-}$] absorbs ∼4–5% water by volume [123] before forming a two phase coexistence. However, because of both force field inaccuracies and finite size effects, computer simulations will most likely not predict coexistence curves exactly. For example, previous computer simulations have predicted 2–3 times higher equilibrium water content in [BMIM${}^{+}$][PF${}_{6}^{-}$] compared to experimental coexistence curves [96]. It is well-known that finite-size effects significantly alter phase-coexistence predictions [124,125], with these effects especially pertinent to liquid-liquid coexistence in which one phase makes up a small fraction of the system. For example, isothermal-isobaric ensemble (e.g., N,P,T) simulations just inside the coexistence region of [BMIM${}^{+}$][PF${}_{6}^{-}$]/water mixtures (>4–5% water) would require the formation of a water-rich phase with a very large interfacial surface area to volume ratio in order to phase separate. This high surface area to volume renders the phase unstable and will shift the coexistence curve to higher water content. It is thus expected that mixtures close to but inside the experimental coexistence region are metastable as a single phase in a computer simulation due to the significant finite size effects. Note that such artifacts are due to inevitable creation of high interfacial surface area and could be avoided using for example, Gibbs ensemble simulations but this is not our focus and clearly beyond the scope of the work [126].

In light of the above discussion, we choose to study hydrophobic IL/water systems that are close to but inside the experimentally determined coexistence region. The upper bound of water content that we study is $\phi_{H_{2}O}$ = 0.13 water fraction by volume, which is roughly 2–3 times experimentally determined saturation [123] but similar to saturation observed in previous computational studies [96]. Based on simulation snapshots, these supersaturated solutions appear to be single phase systems, due to metastability imposed by finite-size effects; we confirm that the force field predicts phase separation for [BMIM${}^{+}$][PF${}_{6}^{-}$] and [BMIM${}^{+}$][TFSI${}^{-}$] at higher water content (e.g., $\phi_{H_{2}O}$ = 0.18–0.25). The benefit of studying these supersaturated solutions is that trends in water structure and water pocket formation are systematically elucidated in spite of any errors in predicted phase behavior from force field errors/finite size effects. In addition, supersaturated solutions are experimentally relevant as they may be stabilized by addition of a small amount of surfactant [127]. We note that based on previous benchmarks, our simulations should be sufficiently long to enable statistical convergence of mixing/phase coexistence in these systems [96,97].

## 3. Results and Discussion

The water nanostructure within IL/water mixtures changes markedly with increasing water content and the nature of these changes depends on the hydrophilicity of the IL. In analogy with prior spectroscopic [52,56] and NMR studies [66,67,71], qualitative structural features can be inferred by characterizing water dynamics; here we focus on the water rotational correlation timescales. In Figure S1, we show rotational correlation functions of water molecules within the five different IL/water mixtures at varying water content. Hydrophilic IL/water mixtures are investigated over systematically varying water content from 0 < $\phi_{H_{2}O}$ < 0.25 volume fraction water and hydrophobic IL/water mixtures are investigated from 0 < $\phi_{H_{2}O}$ < 0.13 volume fraction water, with the latter range (roughly) based on experimental phase coexistence data (see Methods). In this work, we use the terminology “hydrophobic” to refer to the [BMIM${}^{+}$][PF${}_{6}^{-}$] and [BMIM${}^{+}$][TFSI${}^{-}$] ILs, which phase separate at sufficient water content and “hydrophilic” to refer to [BMIM${}^{+}$][BF${}_{4}^{-}$], [BMIM${}^{+}$][NO${}_{3}^{-}$] and [BMIM${}^{+}$][OTf${}^{-}$] which are fully miscible with water [28]. We note that because the melting point of [BMIM${}^{+}$][NO${}_{3}^{-}$] is ≈310 K, all [BMIM${}^{+}$][NO${}_{3}^{-}$] systems are studied at 320 K, with all other systems studied at 300 K (see Methods).

**Figure 1:**
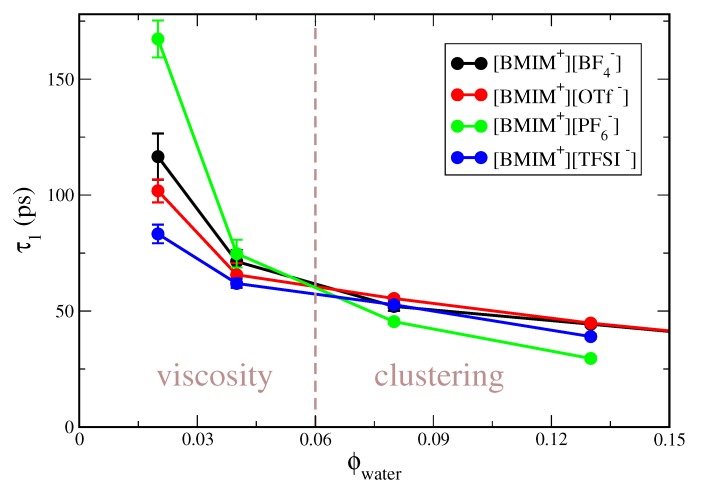
Rotational correlation time $\tau_{1}$ of water molecules as a function of water volume fraction $\phi_{water}$ for [BMIM${}^{+}$][BF${}_{4}^{-}$], [BMIM${}^{+}$][OTf${}^{-}$], [BMIM${}^{+}$][PF${}_{6}^{-}$] and [BMIM${}^{+}$][TFSI${}^{-}$] IL/water mixtures. The vertical dashed line qualitatively indicates the separation of “viscosity” and “clustering” dictated water dynamics. Error bars are given as the difference between predictions separately computed by dividing the trajectory in half.

Figure S1 clearly indicates that concentration-dependent trends in the correlation time differ for the hydrophobic compared to hydrophilic ILs. To better illustrate this effect, the water rotational correlation functions in Figure S1 are integrated to give a characteristic correlation time, $\tau_{1}$. In Figure 1, we plot $\tau_{1}$ as a function of water volume fraction, $\phi_{water}$ for the IL/water mixtures studied at 300 K (i.e., excluding [BMIM${}^{+}$][NO${}_{3}^{-}$]). As indicated by the vertical dashed line in the figure, the water dynamics is dictated by different physical effects at low compared to higher water content. At low water content ($\phi_{water}≲$ 0.05), water dynamics is primarily mediated by the viscosity of the neat IL, corresponding to the “viscosity” region of Figure 1. The viscosity of the pure ILs increases as [BMIM${}^{+}$][TFSI${}^{-}$] < [BMIM${}^{+}$][OTf${}^{-}$] < [BMIM${}^{+}$][BF${}_{4}^{-}$] < [BMIM${}^{+}$][PF${}_{6}^{-}$] [128] and the water rotational relaxation times exactly follow this trend. At the lowest water content studied, $\phi_{water}$ = 0.02, the correlation times are bounded by $\tau_{1}\approx 80$ ps for the lower viscosity [BMIM${}^{+}$][TFSI${}^{-}$] IL and $\tau_{1}\approx 170$ ps for the high viscosity [BMIM${}^{+}$][PF${}_{6}^{-}$] IL. The correlation time of $\tau_{1}\approx 170$ ps for water in [BMIM${}^{+}$][PF${}_{6}^{-}$] at $\phi_{water}$ = 0.02 is nearly two orders of magnitude larger than the corresponding relaxation time in bulk water [129], which approximately mirrors the two order of magnitude higher viscosity of the ILs relative to bulk water.

At higher water content of $\phi_{water}≳$ 0.08, water relaxation dynamics is no longer primarily dictated by the IL viscosity, but rather by the extent of water clustering in the IL/water mixture; this is indicated as the “clustering” regime in Figure 1. For these water concentrations, trends in water relaxation are nearly inverted from the low water content “viscosity” regime, with water exhibiting the fastest relaxation in [BMIM${}^{+}$][PF${}_{6}^{-}$] and the slowest relaxation in the [BMIM${}^{+}$][BF${}_{4}^{-}$] and [BMIM${}^{+}$][OTf${}^{-}$] IL/water mixtures. As we will demonstrate, water mixtures with the hydrophobic [BMIM${}^{+}$][PF${}_{6}^{-}$] and [BMIM${}^{+}$][TFSI${}^{-}$] ILs exhibit a high degree of water clustering, preceding eventual phase separation. Within water clusters, the interior water molecules experience a local water-like environment, exhibiting faster dynamics than water molecules surrounded by the IL ions. We will show that there is significantly less water clustering within the hydrophilic IL/water mixtures and thus the water dynamics in these systems is slower at higher water content due to more complete mixing with the surrounding ions. It is interesting to note that trends in water relaxation dynamics for the hydrophobic IL/water mixtures are qualitatively similar to reported trends in nanoconfined systems, such as reverse micelles [130] or lyotropic liquid crystals (LLCs) [131]. For example, Yethiraj and coworkers [131] found water relaxation times of ∼10 ps $\leq \tau_{1}\leq$ 40 ps for a wide variety of LLCs of different morphology and hydration level. The observation that the water relaxation times span ∼35 ps $\leq \tau_{1}\leq$ 60 ps in [BMIM${}^{+}$][PF${}_{6}^{-}$]/water mixtures at $0.04\leq \phi_{water}\leq 0.13$ water content (Figure 1) and are similar to those in LLC systems supports the notion that water in ionic liquid mixtures can, in many ways, resemble water in nanoconfinement. This correspondence will be further confirmed with subsequent structure/cluster analysis (*vide infra*).

As suggested by the water rotational dynamics (Figure 1), the extent of water clustering within the IL/water mixtures has a substantial impact on the mixture properties. For the remainder of the manuscript, we present and discuss quantitative analysis of the water nanostructure and water clustering for the five IL systems at varying water content. This analysis ranges from local characterization of cluster size and short-range interactions, to a longer range description of water networks provided by percolation analysis. We begin our discussion with characterization of local hydrogen bonding. We compute histograms characterizing the number of water-water hydrogen bonds per molecule within the mixture; a hydrogen bond is defined by O-O distance less than 0.36 nm, O-H distance less than 0.245 nm and H-O-O angle less than 30° [132]. Before discussing all five IL systems, we focus on [BMIM${}^{+}$][BF${}_{4}^{-}$] and [BMIM${}^{+}$][PF${}_{6}^{-}$] water mixtures, as these ILs are strongly hydrophilic and hydrophobic respectively and thus represent limiting behavior.

**Figure 2:**
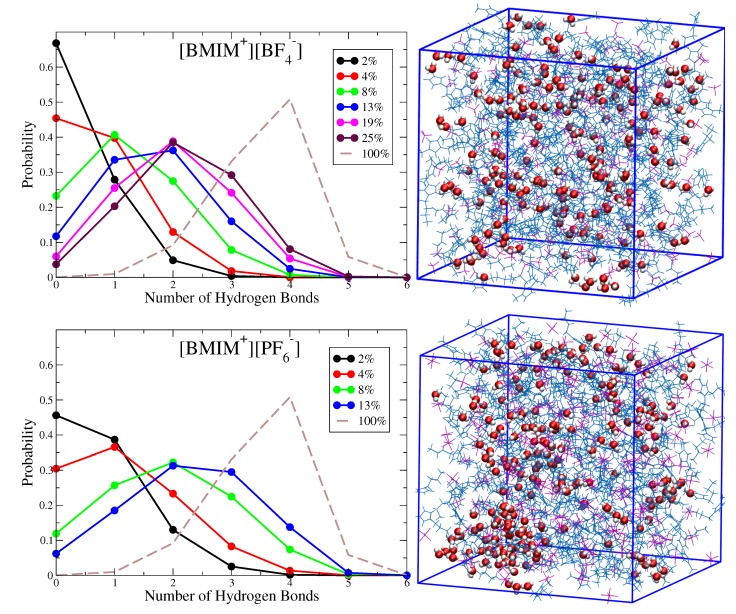
Histograms of number of hydrogen bonds per water molecule for [BMIM${}^{+}$][BF${}_{4}^{-}$] and [BMIM${}^{+}$][PF${}_{6}^{-}$] water mixtures at systematically varying water volume fraction; for comparison, the corresponding histogram for bulk water is shown as dashed line. Simulation snapshots are shown for corresponding systems at $\phi_{water}$ = 0.08 water volume fraction. The statistical uncertainty is on-par with the size of the data point symbols, as estimated from standard deviation of five block averages over the trajectory. The distribution for bulk water was computed from a bulk water simulation with details analogous to those described in Methods.

Figure 2 shows histograms characterizing the probability that a given water molecule will exhibit a specific number of hydrogen bonds with other water molecules in the [BMIM${}^{+}$][BF${}_{4}^{-}$] and [BMIM${}^{+}$][PF${}_{6}^{-}$] water mixtures at different concentrations. Simulation snapshots of the [BMIM${}^{+}$][BF${}_{4}^{-}$] and [BMIM${}^{+}$][PF${}_{6}^{-}$] water mixtures at $\phi_{water}$ = 0.08 are shown alongside the probability distributions for qualitative comparison. These histograms indicate how the water clustering changes with water content in the different ILs. For 4% or lower water content [BMIM${}^{+}$][BF${}_{4}^{-}$] systems, the majority of water molecules are entirely solvated by ions (zero hydrogen bonds), while water-water interactions begin to form at 4% water content in [BMIM${}^{+}$][PF${}_{6}^{-}$] with one hydrogen bond per water being most probable at this concentration. As water content increases beyond 4%, there is increased probability for hydrogen bonding and clustering becomes more substantial in the hydrophobic [BMIM${}^{+}$][PF${}_{6}^{-}$] systems. The simulation snapshots in Figure 2 show the structuring of water in the $\phi_{water}$ = 0.08 IL/water systems. In [BMIM${}^{+}$][PF${}_{6}^{-}$], large, segregated water pockets form within the mixture, and only a small fraction of water molecules (∼10%) are solvated as isolated species (no hydrogen bonds). These water pockets are characterized by water molecules with three or four hydrogen bonds, indicating that local structure approaches a bulk water like coordination environment. The simulation snapshot in Figure 2 indicates that these dynamically changing water pockets in [BMIM${}^{+}$][PF${}_{6}^{-}$] may appear similar to water structures within reverse micelles. In [BMIM${}^{+}$][BF${}_{4}^{-}$], on the other hand, there are no noticeable water pockets and water clustering is restricted to small clusters of water molecules that exhibit between zero and two hydrogen bonds per molecule. This conclusion is consistent with a recent NMR study which concluded that water is mostly dispersed and forms only small water clusters in [BMIM${}^{+}$][BF${}_{4}^{-}$]/water mixtures at low water content [71].

As the water content in [BMIM${}^{+}$][PF${}_{6}^{-}$] approaches 13%, approximately 40 to 50% of the water molecules exhibit between 3 to 4 hydrogen bonds, indicative of water pockets with local bulk-like coordination. Experimentally, this water fraction corresponds to a supersaturated solution that is slightly inside the liquid-liquid, 2-phase coexistence region [123]. However, due to finite size effects as well as any potential force field errors, our simulations indicate a 1-phase mixture at this water content (based on visual inspection) and more rigorous simulation approaches would be needed to determine the precise phase-coexistence predicted by the force field (see Methods). We thus simply interpret this water structural motif as representative of the system“near” the coexistence region/saturation limit. Because the observed water pockets have high surface area to volume ratio, a significant fraction of water is undercoordinated (1–2 hydrogen bonds) at the surface/interface of these water pockets. Convergence to the bulk water hydrogen bond distribution (dashed-line) is thus not expected until the surface to volume ratio of the water pockets is significantly reduced. As indicated by the water rotational dynamics (Figure 1) and simulation snapshots (Figure 2), the water pockets formed within hydrophobic IL/water mixtures near the saturation limit may serve as an excellent prototype for nanoconfined systems such as reverse micelles [61,63,65,69]. The hydrophilic [BMIM${}^{+}$][BF${}_{4}^{-}$] systems, on the other hand, are well-mixed at all water concentrations, with two hydrogen bonds per water molecule being most probable for mixtures of 13 to 25% water volume. Three and four hydrogen bonds per water molecule only becomes probable in [BMIM${}^{+}$][BF${}_{4}^{-}$]/water mixtures when water content is sufficiently high to promote water network percolation on macroscopic lengthscales, with this percolation threshold occurring roughly between ∼19 to 25% water content (*vide infra*). We note that the hydrogen bond histogram for bulk water (Figure 2) interestingly exhibits a finite probability for five hydrogen bonds as has been discussed in previous work [133].

**Figure 3:**
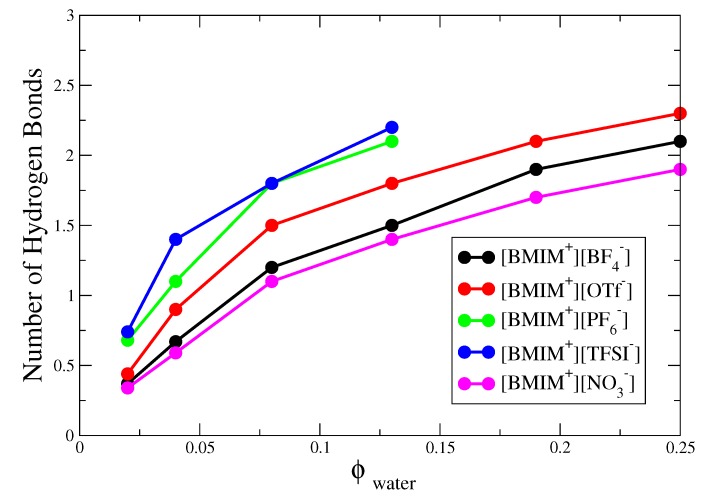
Average number of water-water hydrogen bonds per molecule for IL/water mixtures of $0.02\leq \phi_{water}\leq 0.25$ water volume fraction.

The corresponding hydrogen bond histograms for the [BMIM${}^{+}$][OTf${}^{-}$], [BMIM${}^{+}$][TFSI${}^{-}$] and [BMIM${}^{+}$][NO${}_{3}^{-}$] water mixtures are shown in Figures S2–S4. As indicated by these histograms and corresponding simulation snapshots of 8% water mixtures (Figures S2–S4), the water nanostructure in hydrophilic [BMIM${}^{+}$][NO${}_{3}^{-}$] is very similar to that in the [BMIM${}^{+}$][BF${}_{4}^{-}$] water mixtures, while the nanostructure in hydrophobic [BMIM${}^{+}$][TFSI${}^{-}$] is very similar to that in the [BMIM${}^{+}$][PF${}_{6}^{-}$] mixtures. Like [BMIM${}^{+}$][PF${}_{6}^{-}$], [BMIM${}^{+}$][TFSI${}^{-}$] water mixtures exhibit pronounced water pockets, preceding phase separation at sufficient water content. The [BMIM${}^{+}$][OTf${}^{-}$] water mixtures behave somewhere in the middle compared to the rest of the systems, showing significant clustering and finite probability for tetrahedrally coordinated water, yet these systems are fully miscible at all water content (Videos S1 and S2). To better compare trends, in Figure 3 we plot the average number of hydrogen bonds per water molecule (i.e., integrating the histograms) for the five different types of IL/water mixtures as a function of water content. At 4% or lower water content, the average number of hydrogen bonds is less than 1.5 for all five ILs, indicating mostly isolated water monomers and dimers and only a very small amount of clustering; this is shown schematically by simulation snapshots in Figure S5. As the water content increases to 13%, the hydrophobic [BMIM${}^{+}$][PF${}_{6}^{-}$] and [BMIM${}^{+}$][TFSI${}^{-}$] systems exhibit a significant increase to ∼2–2.5 hydrogen bonds per water molecule, which may be compared to the bulk water limit of ∼3.5 (as computed from our H-bond definition). In the hydrophilic [BMIM${}^{+}$][BF${}_{4}^{-}$] and [BMIM${}^{+}$][NO${}_{3}^{-}$] systems, the increase in hydrogen bond number is less significant and [BMIM${}^{+}$][OTf${}^{-}$] is roughly in the middle in terms of the water clustering. Based on this hydrogen bond criteria, the relative hydrophobicity of ILs depends on water content, with different conclusions inferred from analysis at 2% compared to 4% water content; this conclusion is consistent with previous water absorption free energy calculations [28].

The increasing number of hydrogen bonds with water content is due to a greater number of water molecules existing in large clusters or networks and a smaller fraction of isolated monomer water molecules. The water cluster size distribution provides important information about the onset of long-range network formation [134] and further distinguishes hydrophilic and hydrophobic IL/water mixtures. In Figure S6, we show histograms of water cluster sizes within the IL/water mixtures at 8% water content. For relatively small clusters with ten or fewer water molecules, the distributions look nearly indistinguishable between IL type and all IL/water mixtures exhibit a greater number of isolated monomer water molecules than isolated dimers, trimers or other small cluster motifs. This indicates that the primary difference in water structure between hydrophobic and hydrophilic IL/water mixtures is due to larger clusters/networks formed from N≥10 water molecules. Formation of large clusters/networks intrinsically correlates with substantial fluctuation in cluster size when the system is below the percolation threshold (*vide infra*) [134]. To quantify fluctuations in cluster size and formation of larger water networks, we compute the “weight averaged cluster size”, $\langle n_{w}\rangle$, which has been previously defined and utilized for water cluster analysis [134]. Essentially, this metric reflects the average cluster size computed from a weight function equal to the fraction of water molecules within specific size clusters (Equation (S3)). Additionally, $\langle n_{w}\rangle$ can be interpreted as a variance of cluster size, for example, $\langle n^{2}\rangle$, for the distribution of different sized clusters and thus provides information on cluster size fluctuations [134]. In Figure S7, we show $\langle n_{w}\rangle$ computed for the five different IL/water mixtures over the range of water content. There is direct correlation between the magnitude of $\langle n_{w}\rangle$ and the hydrophobicity of the IL; [BMIM${}^{+}$][NO${}_{3}^{-}$] exhibits the smallest $\langle n_{w}\rangle$ at a given water content (smallest clusters), while the largest values of $\langle n_{w}\rangle$ occur for [BMIM${}^{+}$][PF${}_{6}^{-}$]/water mixtures (largest clusters). This analysis clearly indicates that large water clusters and networks are formed in the hydrophobic IL/water mixtures at 8% and greater water content and correspondingly there are large fluctuations in cluster size.

**Figure 4:**
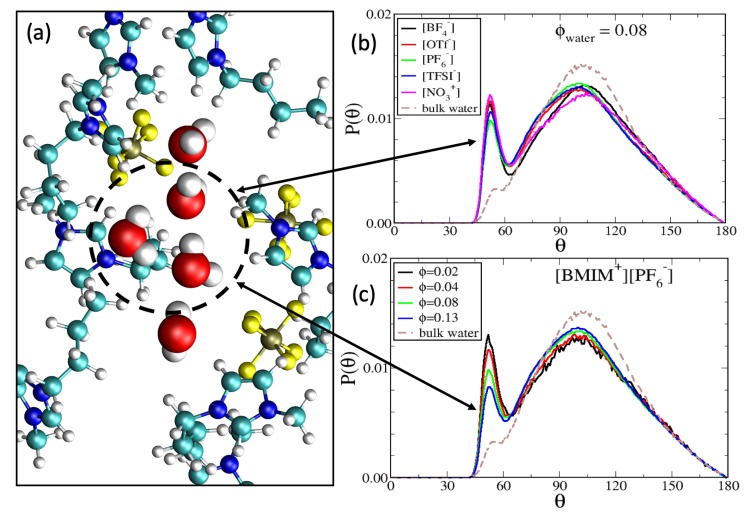
(**a**) Simulation snapshot of water triangle motif formed within [BMIM${}^{+}$][PF${}_{6}^{-}$]/water mixture at $\phi_{water}$ = 0.08 water content. Probability distribution of O-O-O angles for water trimers within (**b**) IL/water mixtures at $\phi_{water}$ = 0.08 water content and (**c**) [BMIM${}^{+}$][PF${}_{6}^{-}$] IL/water mixtures at varying water content. The distribution for bulk water was computed from a bulk water simulation with details analogous to those described in Methods.

To further elucidate the local coordination environment of water, we compute the O-O-O angle distribution of water trimers within the IL/water mixtures and the corresponding tetrahedral order parameter. The tetrahedral order parameter “*q*” is defined as

(1) $$q=1-\frac{9}{4}{\left(cos\theta +\frac{1}{3}\right)}^{2}$$

and is a measure of how the local coordination reflects a tetrahedral geometry [135]. The angle θ is the O-O-O angle of water trimers and note that θ = 109.5° corresponds to *q* = 1. As indicated by Figure S6, not all water molecules form trimers; trimers may be present within water clusters composed of three molecules or more, but there are also isolated monomers and dimers. To restrict our analysis to neighboring molecules, we define a trimer as three water molecules with oxygen-oxygen distance (from the central molecule) less than 0.36 nm. In Figure 4b, we show the O-O-O angle distribution of water trimers in the five different IL/water mixtures at $\phi_{water}$ = 0.08 water content, along with the corresponding reference distribution for bulk water. The primary feature of the distribution is the broad peak centered at the tetrahedral coordination angle (θ = 109.5°), reminiscent of the bulk water environment. However, an important feature within the IL/water mixtures is the narrow second peak at the shoulder of the bulk distribution, centered at θ = 50–60°. While this second peak is present in the bulk water distribution, it is significantly more pronounced for the water trimers within the IL/water mixtures. For bulk water, this small peak has been attributed to triangular ring-like structures [136,137,138], as indicated by the θ = 50–60° angle being close that of an equilateral triangle; we thus refer to this as a ‘water triangle’ peak.

Because water triangles are largely incompatible with the tetrahedral orientation of bulk water, the water triangle peaks in the distributions of Figure 4 indicate water aggregates that deviate from bulk-like structures. In Figure 4a, we show a simulation snapshot of a water triangle motif within the [BMIM${}^{+}$][PF${}_{6}^{-}$]/water mixture at $\phi_{water}$ = 0.08 water content. The snapshot depicts four water molecules that form a wire in a linear chain of hydrogen bonds and the water triangle is formed when an outside water molecule donates one hydrogen bond to each of two adjacent water molecules in the water wire. From the O-O-O angle distributions in Figure 4b, it is seen that water triangles are formed with different probability within the different ILs. For $\phi_{water}$ = 0.08 water content, the water triangle peak is greater in magnitude for hydrophilic [BMIM${}^{+}$][BF${}_{4}^{-}$] and [BMIM${}^{+}$][NO${}_{3}^{-}$] ILs than it is for hydrophobic [BMIM${}^{+}$][PF${}_{6}^{-}$] and [BMIM${}^{+}$][TFSI${}^{-}$] ILs. This indicates that water triangles are more prevalent in hydrophilic ILs due to more complete IL/water mixing, while tetrahedral water coordination is more prevalent in hydrophobic ILs which contain bulk-like water pockets, as qualitatively indicated by snapshots in Figure 2. It is important to note that Figure 4 is not an *absolute* probability distribution but rather is a *conditional* probability distribution; it provides the probability of a specific angular orientation given that a trimer is formed. The absolute probability of forming trimers is indicated by Figures S6 and S7.

Figure 4c shows the trimer angle distributions in [BMIM${}^{+}$][PF${}_{6}^{-}$]/water mixtures at varying water content, 0 < $\phi_{H_{2}O}$ < 0.13. For these systems, the tetrahedral order parameter increases with water content as *q* = 0.44, *q* = 0.46, *q* = 0.49 and *q* = 0.51 for water fractions $\phi_{H_{2}O}$ = 0.02, $\phi_{H_{2}O}$ = 0.04, $\phi_{H_{2}O}$ = 0.08 and $\phi_{H_{2}O}$ = 0.13 respectively. These order parameters may be compared to the value of *q* = 0.63 for bulk water [137,138,139] and indicate that the local water structure becomes more bulk-like for hydrophobic IL/water mixtures with increasing water content. The angle distribution in Figure 4c directly correlates with the hydrogen bond distribution in Figure 2: Water molecules in triangular motifs exhibit two hydrogen bonds each, or three if they simultaneously participate in wire structures (Figure 4). As seen in Figure 2, increasing water content results in a shift from 2–3 hydrogen bonds to 3–4 hydrogen bonds per water molecule within a pocket, correlating to water triangles being substituted for tetrahedral water.

**Figure 5:**
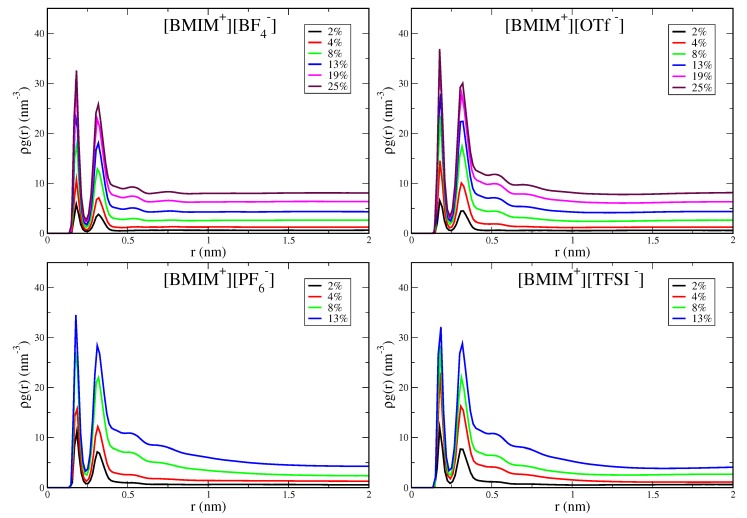
Pairwise probability distribution for water computed from oxygen-hydrogen distances for [BMIM${}^{+}$][BF${}_{4}^{-}$], [BMIM${}^{+}$][OTf${}^{-}$], [BMIM${}^{+}$][PF${}_{6}^{-}$] and [BMIM${}^{+}$][TFSI${}^{-}$] IL/water mixtures. Distributions are normalized by the density of water molecules.

With increasing water cluster and water pocket formation, the IL/water mixtures become more heterogeneous and eventually phase separate in the case of the hydrophobic IL systems. It is thus important to quantify the concentration onset and the spatial lengthscale of heterogeneity within the IL/water mixtures. The pairwise correlation functions, in particular the shape and asymptotic behavior, provide an indication of this heterogeneity onset. In Figure 5, we show water-water pairwise distribution functions for the [BMIM${}^{+}$][BF${}_{4}^{-}$], [BMIM${}^{+}$][OTf${}^{-}$], [BMIM${}^{+}$][PF${}_{6}^{-}$] and [BMIM${}^{+}$][TFSI${}^{-}$] IL/water mixtures; the analogous graph for [BMIM${}^{+}$][NO${}_{3}^{-}$] is shown in Figure S9. Rather than plotting the radial distribution function, g(r), we plot ρg(r) because the water concentration is changing for the mixtures (ρ is the density of water molecules). The two peaks at short range reflect local hydrogen bonding structure, with the first hydrogen bond peak at 0.18 nm for all IL/water mixtures, as expected. The medium to long-range region of these correlation functions reflects the differing onset of heterogeneity in the mixtures. The hydrophilic IL mixtures, [BMIM${}^{+}$][BF${}_{4}^{-}$] (Figure 5) and [BMIM${}^{+}$][NO${}_{3}^{-}$] (Figure S9), exhibit flat asymptotic behavior in the distributions for r ≳ 0.75 nm. This is indicative of a homogeneous, well-mixed system with water molecules uniformly dispersed. In contrast, the hydrophobic ILs, [BMIM${}^{+}$][PF${}_{6}^{-}$] and [BMIM${}^{+}$][TFSI${}^{-}$], display distinctly different behavior and exhibit significant curvature in the distribution tails at medium to long range. This curvature reflects the heterogeneity lengthscale within the mixtures and indicates the onset of water domain formation preceding eventual phase separation. Note that integration of the pairwise distribution functions gives the water coordination number, N${}_{coord}$, which when computed using an integration cutoff value just beyond the second peak, gives a very similar profile (Figure S8) to the average hydrogen bond numbers in Figure 3.

While all IL/water systems are well mixed at low water content (≲4% ), the curvature in the pairwise distribution tails for hydrophobic ILs (Figure 5) at ∼8–13% water content indicates significant water pocket formation and structural heterogeneity over nanometer lengthscales. The lack of decay of the correlation functions over 1–2 nm lengthscales for the [BMIM${}^{+}$][PF${}_{6}^{-}$] and [BMIM${}^{+}$][TFSI${}^{-}$] systems indicates that the mixtures are very heterogeous with formation of large water pockets for the higher water content systems. The [BMIM${}^{+}$][OTf${}^{-}$] water mixtures show interesting, intermediate behavior, with consistent but subtle structural heterogeneity over ∼1 nm lengthscales at water content above 8%. The nature of water clustering giving rise to the structural heterogeneity in [BMIM${}^{+}$][OTf${}^{-}$] is qualitatively seen in the simulation snapshot in Figure S2. Despite this structural heterogeneity, [BMIM${}^{+}$][OTf${}^{-}$] is a hydrophilic IL as it is miscible with water at all concentrations. The pairwise distribution functions (Figure 5) thus enable definitive classification into hydrophilic and hydrophobic ILs: All hydrophilic IL/water mixtures, [BMIM${}^{+}$][BF${}_{4}^{-}$], [BMIM${}^{+}$][NO${}_{3}^{-}$], [BMIM${}^{+}$][OTf${}^{-}$], decay to their asymptotic values at intermediate range (∼1 nm), whereas the hydrophobic systems, [BMIM${}^{+}$][PF${}_{6}^{-}$] and [BMIM${}^{+}$][TFSI${}^{-}$] exhibit significant curvature in their distributions beyond 1 nm. In lieu of more rigorous phase-coexistence simulations, these distributions qualitatively indicate the onset of phase separation of hydrophobic IL/water mixtures as reflected in the long-range heterogeneity.

**Figure 6:**
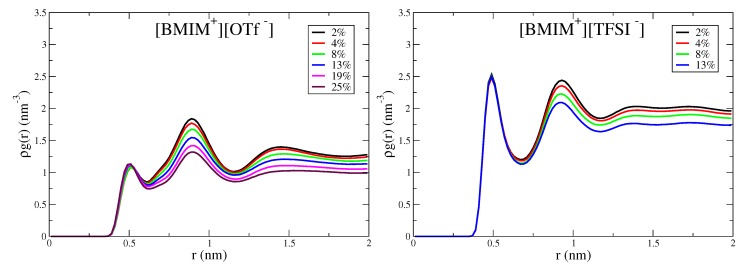
Anion/anion pairwise distributions computed from carbon atoms of -CF${}_{3}$ groups for [BMIM${}^{+}$][OTf${}^{-}$] and [BMIM${}^{+}$][TFSI${}^{-}$] water mixtures. Distributions are normalized by the density of anion carbon atoms.

The hydrophobic -CF${}_{3}$ groups of the OTf${}^{-}$ anions give rise to the subtle structural heterogeneity observed in Figure 5, despite [BMIM${}^{+}$][OTf${}^{-}$] being fully miscible with water. To better characterize this hydrophobic effect, we compute anion-anion correlation functions based on the distance between -CF${}_{3}$ groups. Figure 6 shows the corresponding correlation functions for the [BMIM${}^{+}$][OTf${}^{-}$] mixtures, as well as [BMIM${}^{+}$][TFSI${}^{-}$] for comparison, as the TFSI${}^{-}$ anions also contain -CF${}_{3}$ groups. Both ILs show short range peaks at ∼0.5 nm, corresponding to close-packed, hydrophobic CF${}_{3}$ groups within the mixtures. Interestingly, the magnitude of this close contact, hydrophobic-packing peak does not significantly change with water content. This means that the number of hydrophobic associations is not changing with water content, as this number is given by integrating ρg(r). For [BMIM${}^{+}$][OTf${}^{-}$]/water mixtures, this is a somewhat surprising result, considering that the ions and water mix at all concentrations. We conclude, therefore, that the hydrophobic effect that drives association of -CF${}_{3}$ is present with and without water. In tandem with the hydrophobic effect mediated by water, the ions themselves mediate a similar hydrophobic association driving force that favors association of polar and charged moities within the IL. This hydrophobic effect promotes anion/anion packing (-CF${}_{3}$ groups), which has been previously observed by Schwenzer et al. for neat [BMIM${}^{+}$][OTf${}^{-}$] IL [140]. The concentration invariance of the hydrophobic peak in Figure 6 implies that the strength of the hydrophobic effect is similar as mediated by water and ILs, which is an interesting conclusion.

**Figure 7:**
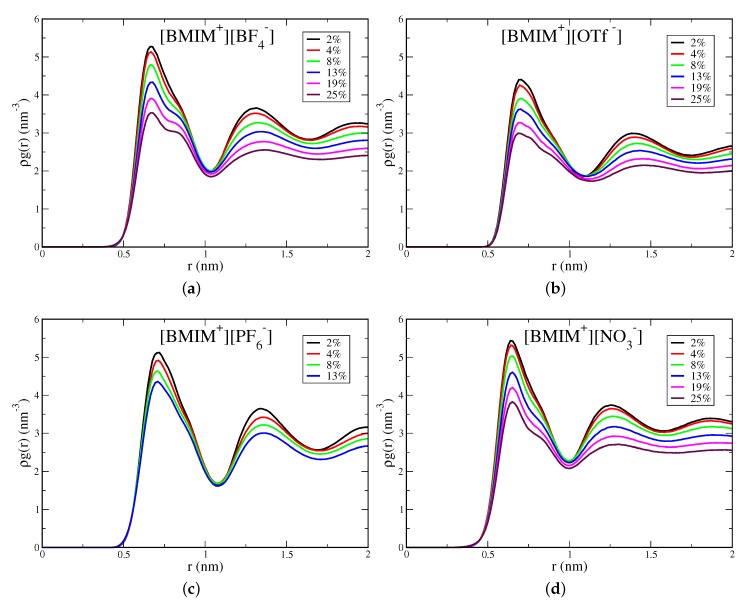
Anion-anion pairwise distributions computed from anion center of mass for (**a**) [BMIM${}^{+}$][BF${}_{4}^{-}$], (**b**) [BMIM${}^{+}$][OTf${}^{-}$], (**c**) [BMIM${}^{+}$][PF${}_{6}^{-}$] and (**d**) [BMIM${}^{+}$][NO${}_{3}^{-}$] IL/water mixtures. Distributions are normalized by the density of anions.

Water screening of ion pairs is an important interaction motif that affects the extent of clustering in the IL/water mixtures. Because the anions are smaller with more concentrated charge, prominent interactions are expected to involve water molecule(s) screening anion repulsion at close distance. To analyze these interactions, we compute anion/anion pairwise distribution functions ρg(r), based on the anion center of mass. These distributions are shown in Figure 7 for [BMIM${}^{+}$][BF${}_{4}^{-}$], [BMIM${}^{+}$][OTf${}^{-}$], [BMIM${}^{+}$][PF${}_{6}^{-}$] and [BMIM${}^{+}$][NO${}_{3}^{-}$] and in Figure S10 for [BMIM${}^{+}$][TFSI${}^{-}$]/water mixtures. All systems show a peak at ∼0.6–0.7 nm lengthscale, which reflects the characteristic anion separation distance in the corresponding neat IL [111]. This peak is the closest anion separation distance in the mixtures and is present at all concentrations. For neat ILs, it has been shown that this 0.6–0.7 nm lengthscale corresponds to oscillations of shells of counterions within the liquid [111]. It is notable that addition of water does not give rise to a closer distance anion/anion peak, which might be expected for solvent separated anion pairs. Potential of mean force (PMF) calculations have demonstrated that solvent-separated anion pairs are unstable at high dilution for BF${}_{4}^{-}$ and NO${}_{3}^{-}$ anions and are slightly stable for the more hydrophobic PF${}_{6}^{-}$, OTf${}^{-}$ and TFSI${}^{-}$ anions [28].

For the hydrophilic IL/water mixtures, this anion peak is split into a larger separation distance peak/shoulder that emerges at higher water content, as is seen in Figure 7 for [BMIM${}^{+}$][BF${}_{4}^{-}$], [BMIM${}^{+}$][NO${}_{3}^{-}$] and somewhat for [BMIM${}^{+}$][OTf${}^{-}$]. The splitting of these new “doublet” peaks is ∼0.2 nm, which is approximately a hydrogen bond distance. Analysis of anion/water pair distributions confirms that hydrogen bonds at ∼0.2 nm distance are formed between oxygen atoms of sulfonate anion groups and hydrogen atoms of water (Figure S11). These longer distance peaks/shoulders in the doublet are thus at ∼0.8–0.9 nm distances and reflect longer distance anion-anion separation than in the bulk IL due to the formation of a hydrogen bond between water and one of the anions. They do not, however, reflect single water molecule separated anion pairs, which would occur at closer distances [28]. This effect is visible in [BMIM${}^{+}$][OTf${}^{-}$] but the peaks lack structure and are more diffuse compared to the doublet peaks in [BMIM${}^{+}$][BF${}_{4}^{-}$] and [BMIM${}^{+}$][NO${}_{3}^{-}$]. For the hydrophobic [BMIM${}^{+}$][PF${}_{6}^{-}$] and [BMIM${}^{+}$][TFSI${}^{-}$] ILs, there is no water-mediated splitting of the characteristic anion IL peak (Figure 7 and Figure S10). Furthermore, for the hydrophobic ILs, the change in peak height with water content is less pronounced than for the hydrophilic ILs.

Figure 7 indicates that the extent of modulation of anion-anion interactions by water is directly correlated with the hydrophilicity of the IL and not with the strength of local anion-water interactions. Similar hydrogen bonds are observed between sulfonate groups and water for both OTf${}^{-}$ and TFSI${}^{-}$ based ILs, yet Figure 7 and Figure S10 show that water is more incorporated into the structure of [BMIM${}^{+}$][OTf${}^{-}$] than in [BMIM${}^{+}$][TFSI${}^{-}$]. Previous work demonstrated that water screening of anion repulsion contributes a significant energetic driving force for mixing of hydrophilic but not hydrophobic ILs with water [28]. Because of the positive charge of the cation, protons on the imidazolium ring are potential hydrogen bond donors, and it is possible that such cation-water interactions could play a role in influencing IL/water structure. We have computed cation/water pairwise distribution functions between protons on the imidazolium ring and water oxygen atoms, with these distributions shown in Figure S12. We find hydrogen/oxygen distances of ∼0.28 nm between cation and water, which is somewhat larger separation than a typical hydrogen bond. This rather weak correlation indicates that cation/water interactions are likely not a primary structure-directing driving force, consistent with prior interpretation [25].

Our final analysis concerns the percolation of water networks within the IL/water mixtures. We have quantified the microscopic (nanometer lengthscale) water structure/clustering within IL/water mixtures and percolation analysis provides an important connection with the structure over macroscopic lengthscales. Percolation analysis involves two steps: First, some connectivity metric must be defined between adjacent percolating units; for water, we make the natural choice that two water molecules are connected if and only if there is a hydrogen bond between them. Next, some algorithm (usually recursive [122]) must be applied to check whether there exists a continuous network of connected (hydrogen bonded) water molecules that entirely spans the system length and is connected with itself through the periodic boundaries of the simulation box. If such a network exists, then it is said to percolate. Percolating networks may form and break during the course of the simulation and the probability of finding the system with a percolating network is defined as $R\left(\phi ,V\right)$. This probability obviously depends on the amount of water in the system (ϕ is the volume fraction of water) but also depends on the system size (V, the volume of the simulation box).

**Figure 8:**
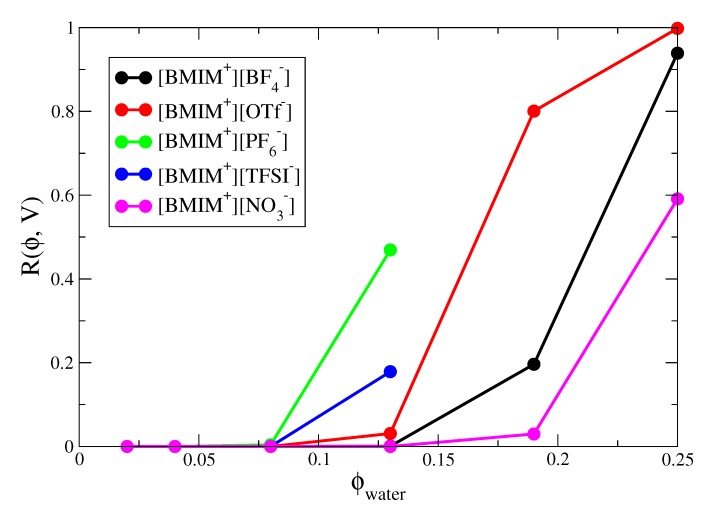
Percolation probability $R\left(\phi ,V\right)$ as a function of water volume fraction, $\phi_{water}$, for IL/water mixtures.

Percolation analysis enables determination of the minimal water content required such that water networks are connected over macroscopic lengthscales. In IL/water mixtures, long range connectivity of water networks is important for ion transport and related processes. Figure 8 shows the percolation probabilities of the IL/water mixtures as a function of water volume fraction. The percolation probabilities show the expected sigmoidal dependence on water volume fraction and the sigmoidal curve would approach a “step function” in the limit of infinite system size [141,142]. The percolation threshold is approximately located at the inflection point of the sigmoidal curves and represents the specific concentration at which an infinite system undergoes a transition from zero to unit probability percolation. From the curves in Figure 8, we estimate the percolation threshold to occur at $\phi_{water}$ = 0.17, $\phi_{water}$ = 0.22 and $\phi_{water}$ = 0.25 for [BMIM${}^{+}$][OTf${}^{-}$], [BMIM${}^{+}$][BF${}_{4}^{-}$] and [BMIM${}^{+}$][NO${}_{3}^{-}$] respectively. The hydrophobic ILs would exhibit lower percolation thresholds but because these systems phase separate at higher water content, more rigorous analysis (i.e., accounting for finite size effects) would be required to determine whether the percolation threshold lies within the miscibility region. The trend in percolation threshold for the hydrophilic ILs correlates with the average number of water-water hydrogen bonds formed in the IL/water mixtures (Figure 3), with water molecules in [BMIM${}^{+}$][OTf${}^{-}$] forming more hydrogen bonds than those in [BMIM${}^{+}$][NO${}_{3}^{-}$] at a given concentration. Also, and as noted previously [134], the percolation probabilities correlate well with the weight averaged cluster size ($n_{w}$) distributions of the IL/water mixtures (Figure S7), indicating the close connection between percolation and fluctuations. It is interesting to note that the [BMIM${}^{+}$][OTf${}^{-}$] system which exhibits the lowest concentration percolation threshold of the three hydrophilic ILs, is also composed of anions with the most amphiphilic character. The hydrophobic -CF${}_{3}$ groups of the triflate anions exhibit a tendency to cluster (Figure 6), while the sulfonate groups strongly coordinate water molecules (Figure S11). The paradigm of facile water percolation within IL water mixtures composed of amphiphilic anions may be an important design motif for membrane materials.

## 4. Conclusions

The behavior of water in nanoconfined environments has long been a subject of interest to chemists and biochemists. Due to their amphiphilic character and strong Coulombic interactions, ionic liquids form mixtures with water that exhibit a variety of water networks, with water structures often resembling those in nanoconfined systems. We have thoroughly investigated five ILs at systematically varying water content to illustrate the dependence of water network structure on both the amount of water and the hydrophobicity of the IL. In these systems, the water structure ranges from water pockets with bulk-like coordination in hydrophobic ILs, to isolated molecules, connected chains or small water clusters in more hydrophilic IL mixtures at lower water content, to fully percolating and connected water networks at 15–25% water volume fraction in hydrophilic IL/water mixtures. The similarity to water in nanoconfined systems is revealed through analysis of both structure and dynamics. Water pockets in hydrophobic [BMIM${}^{+}$][PF${}_{6}^{-}$] and [BMIM${}^{+}$][TFSI${}^{-}$] ILs span nanometer distances and consist of water molecules with 3–4 hydrogen bonds and tetrahedral coordination structure and this structure is reminiscent of water in reverse micelles. Furthermore, we have shown that water molecules in hydrophobic IL/water mixtures at (supersaturated) 8 to 13% water volume content exhibit very similar rotational relaxation dynamics to water in a variety of (previously characterized) lyotropic liquid crystals.

From a pragmatic perspective, tuning water networks for a desired application may involve choosing the ionic liquid that is “just right”; too hydrophobic ILs may lead to immiscibility, while strongly hydrophilic ILs may result in too well-solvated and isolated water molecules/clusters. Furthermore, because the properties strongly depend on water content, various types of IL/water mixtures may be optimal for different concentration ranges. This situation is clearly illustrated by our percolation analysis. For membrane applications involving mass or ion transport, water networks should be connected over macroscopic lengthscales or, in other words, the mixture should be above the percolation threshold. Also, in many cases, the mixture should be miscible and should not phase-separate. Of the five ILs studied, [BMIM${}^{+}$][OTf${}^{-}$] exhibits intermediate hydrophobicity, being fully miscible with water over full concentration range, yet exhibiting signatures of hydrophobic aggregation and nanoscale domain formation. The water percolation threshold occurs at lower water content for [BMIM${}^{+}$][OTf${}^{-}$] compared to the other hydrophilic ILs, indicating that it may be an ideal choice for mixtures used in membrane applications. The triflate anion evidently exhibits optimal amphiphilic character to facilitate water percolation, with water networks avoiding hydrophobic domains of -CF${}_{3}$ groups, and well-solvated by the sulfonate groups of the anion.

Like water, ionic liquids intrinsically mediate hydrophobic forces, which is an important factor enabling the significant tunability of water structures within IL/water mixtures. The tendency for hydrophobic aggregation results from both energetic and entropic driving forces when unlike interactions are present in a system. While oil and water is the typical case, neat ionic liquids intrinsically exhibit unlike interactions; for example the strong electrostatic interactions between the ionic and dipolar imidazolium ring of cations and sulfonate groups of anions differ markedly from the interactions of the aliphatic cation chains or -CF${}_{3}$ groups on anions. Rationalizing the nanostructure of IL/water mixtures thus involves consideration of hydrophobic forces intrinsic to both water as well as the neat IL itself. The complementary nature of these hydrophobic forces was illuminated through analysis of the pairwise correlation of hydrophobic -CF${}_{3}$ groups as a function of water content, for [BMIM${}^{+}$][OTf${}^{-}$] and [BMIM${}^{+}$][TFSI${}^{-}$] IL/water mixtures. In absence of hydrophobic forces intrinsic to the neat IL, one would expect increased correlation between -CF${}_{3}$ groups with increasing water content due to hydrophobic association forces imposed by water. However this is not the case but rather it was observed that -CF${}_{3}$ correlation is largely invariant to water content, for both [BMIM${}^{+}$][OTf${}^{-}$] and [BMIM${}^{+}$][TFSI${}^{-}$]. Evidently, hydrophobic association forces within the bulk IL are similar to those imposed by the presence of water in terms of influence on this pairwise correlation. We look forward to future work that explores parallels between these interaction motifs in IL/water mixtures with those in biochemical systems.

## Figures and Tables

**Table 1:** Number of water molecules in ionic liquid (IL)/water systems for specified water volume fraction, $\phi_{H_{2}O}$. All systems contain 220 ion pairs.

	$\phi_{H_{2}O}=0.02$	$\phi_{H_{2}O}=0.04$	$\phi_{H_{2}O}=0.08$	$\phi_{H_{2}O}=0.13$	$\phi_{H_{2}O}=0.19$	$\phi_{H_{2}O}=0.25$
[BMIM${}^{+}$][BF${}_{4}^{-}$]	45	92	200	350	550	750
[BMIM${}^{+}$][NO${}_{3}^{-}$]	43	88	184	324	509	693
[BMIM${}^{+}$][OTf${}^{-}$]	54	110	237	417	654	892
[BMIM${}^{+}$][PF${}_{6}^{-}$]	50	102	218	385	—	—
[BMIM${}^{+}$][TFSI${}^{-}$]	71	145	311	548	—	—

## Supplementary Materials

The following are available online at http://doi.org/10.5281/zenodo.3585693.
